# Correction: Application of the cyberinfrastructure production function model to R1 institutions

**DOI:** 10.3389/frma.2026.1843420

**Published:** 2026-04-27

**Authors:** Preston M. Smith, Jill Gemmill, David Y. Hancock, Brian W. O'Shea, Winona Snapp-Childs, James Wilgenbusch

**Affiliations:** 1 Rosen Center for Advanced Computing, Purdue University, West Lafayette, IN, United States; 2 Research Computing and Data, Clemson Computing & Information Technology, Clemson University, Clemson, SC, United States; 3 Research Technologies Division, Office of the Vice President for Information Technology, Indiana University, Bloomington, IN, United States; 4 Institute for Cyber-Enabled Research, Michigan State University, East Lansing, MI, United States; 5 Department of Computational Mathematics, Science, and Engineering, Michigan State University, East Lansing, MI, United States; 6 Department of Physics and Astronomy, Michigan State University, East Lansing, MI, United States; 7 Facility for Rare Isotope Beams, Michigan State University, East Lansing, MI, United States; 8 Pervasive Technology Institute, Indiana University, Bloomington, IN, United States; 9 Research Computing, Research & Innovation Office, University of Minnesota, St. Paul, MN, United States

**Keywords:** cyberinfrastructure, ROI, HPC, economics, value proposition, research computing and data, RCD

Equation 7 in **Section 3.6** was erroneously given as: Salaries = $3,400 x N_pub._ The correct equation is *Salaries* = $340 × N_pub_

A number: $3,400, was incorrectly stated in **Section 3.6**, paragraph 2. The correct number should be $340.

A correction has been made to **Section 3.6**, paragraph 2:

The average ratio of TeraFLOPs of capacity to publication output is 1.34 TeraFLOPs per publication reported in Scopus (**Benchmark Q1**), and the average salary cost per earned doctorate is $340 (**Benchmark Q2**).

The original version of this article has been updated.

